# Supplementary material for: The Effect of the Position of a Phenyl Group on the Luminescent and TNP-Sensing Properties of Cationic Iridium(III) Complexes
Source: Sensors (Basel). 2025 Jan 30;25(3):839. doi: 10.3390/s25030839 (PMC11821259; doi:10.3390/s25030839)
Supplement: Supplementary file 1 [file sensors-25-00839-s001.zip › sensors-3406675-supplementary.pdf]

Supplementary data for

Effect of the position of a phenyl group on the luminescent and TNP-sensing properties of cationic iridium(III) complexes

Xiaoran Yang, Jiahao Du, Rui Cai, Chun Liu *

State Key Laboratory of Fine Chemicals, Frontier Science Center for Smart Materials, School of Chemical Engineering, Dalian University of Technology, Linggong Road 2, Dalian 116024, China; E-mail: cliu@dlut.edu.cn.

Contents

Materials and instruments *S1*

Synthesis and characterization *S2-S4*

Preparation and Detection of TNP Samples *S4*

Photophysical and AIPE properties *S4*

Detection of TNP *S5*

Sensing Mechanism *S5-S6*

NMR spectra and HRMS of **1**, **2**, and **3** *S7-S11*

Materials and instruments

All starting materials were purchased from commercial sources and used without further purification. The ^{1}H NMR spectra were recorded at Bruker AVANCE III 500. The ^{13}C NMR spectra was recorded at BRUKER AVANCE NEO 400MHz. Mass spectra were obtained on a Synapt G2-Si HDMS and Agilent G6230B mass spectrometer. Absorption spectra were recorded using Agilent Cary 100 UV-vis spectrophotometer. The emission spectra were recorded with a Hitachi F-7100 fluorescence spectrophotometer. Lifetime decay traces were tested using a transient fluorescence spectrometer FLS1000. The dynamic light scattering (DLS) was measured

on Malvern ZS90.

Synthesis and characterization

Synthesis of ligands L1-L3

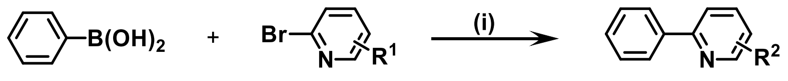

m1 : R^1 = 3-Br; m2 : R^1 = 4-Br; m3 : R^1 = 5-Br

L1 : R^2 = 3-Ph; L2 : R^2 = 4-Ph; L3 : R^2 = 5-Ph

(i) $Pd(OAc)_2$, K_2CO_3, EtOH/H_2O 3:1 (v/v), 80°C, air.

Figure S1. Synthesis route of the cyclometalating ligands **L1-L3**.

The cyclometalating ligands were synthesized using the Suzuki-Miyaura reaction catalyzed by $Pd(OAc)_2$ developed by our group. To a double-necked flask, 0.5 mmol of 2-bromopyridine derivative, phenylboronic acid (3.0 equiv.), potassium carbonate (2.0 equiv.), and palladium acetate (1.5% equiv.) were added sequentially in air, followed by 8 mL of a mixed ethanol-water solution with a volume ratio of 3:1, and the reaction was magnetically stirred for 10-50 min at 80°C. At the end of the reaction, it was extracted with saturated saline and dichloromethane, and the organic phases were combined and concentrated under reduced pressure. The cyclometalating ligands **L1-L3** were prepared by column chromatography with petroleum ether/ethyl acetate (30:1) as eluent.

Synthesis and characterization of the complexes 1-3

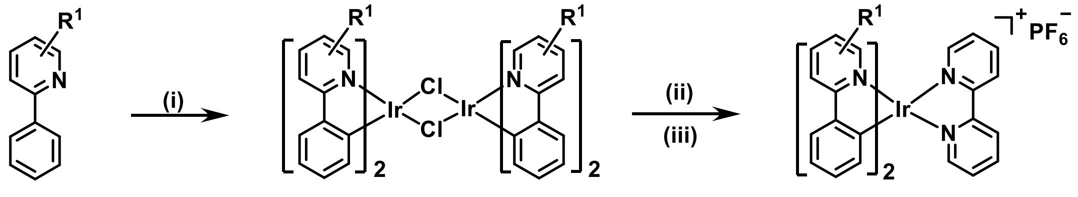

1 : R^1 = 3-Ph; 2 : R^1 = 4-Ph; 3: R^1 = 5-Ph

(i) $IrCl_3{\cdot}3H_2O$, $EtOCH_2CH_2OH/H_2O$, 3:1 (v/v), 120 °C, N_2, 24 h.
(ii) 2,2'-Bipyridine, $EtOCH_2CH_2OH$, 120 °C, N_2, 24 h. (iii) KPF_6, RT, 3 h.

Figure S2. Synthesis route of the complexes **1-3**.

In the first step, $IrCl_3{\cdot}3H_2O$ (0.20 mmol) was added to a 25 mL double-necked flask,

and then cyclometalating ligand (0.5 mmol), 12 mL of a mixed solution of ethylene glycol monoethyl ether/water in the ratio of 3:1 by volume, was added sequentially, and the reaction was carried out for 24 h at 120°C under nitrogen protection with nitrogen displacement for three times. At the end of the reaction, it was cooled to room temperature and concentrated under reduced pressure to give the intermediate.

In the second step, the above intermediate was transferred to a 50 mL single-necked round-bottomed flask and 2,2'-bipyridine (0.6 mmol) and ethylene glycol monoethyl ether (8 mL) were added. The reaction was carried out under nitrogen atmosphere at 120°C for 24 h. After the reaction, the mixture was cooled to room temperature, and an excess of saturated aqueous solution of KPF_6 (15 equiv.) was added and stirred at room temperature for 3 h. The resulting mixture was extracted with saturated saline and dichloromethane, and the organic phases were combined and concentrated to obtain the crude product. The target product was isolated and purified using dichloromethane as eluent.

1, yellow solid, 130.1 mg, yield: 68%. ^{1}H NMR (400 MHz, DMSO-d_6) δ 8.93 (d, J = 8.2 Hz, 2H), 8.31 (t, J = 7.8 Hz, 2H), 7.86 (d, J = 5.1 Hz, 2H), 7.80 - 7.67 (m, 6H), 7.63 - 7.42 (m, 8H), 7.31 (s, 2H), 7.23 - 7.13 (m, 2H), 6.82 (t, J = 7.2 Hz, 2H), 6.66 - 6.52 (m, 4H), 6.30 (d, J = 7.5 Hz, 2H). ^{13}C NMR (125 MHz, DMSO-d_6) δ 163.98, 155.27, 152.27, 149.64, 148.52, 144.12, 141.34, 139.77, 138.91, 136.58, 131.07, 129.55, 129.18, 128.61, 125.19, 123.21, 121.15. HRMS (Synapt G2-Si HDMS, *m/z*): calcd for $C_{44}H_{32}N_4Ir$ [M-PF$_6$]$^+$ 809.2251, found 809.2251.

2, yellow solid, 132.6 mg, Yield: 70%. ^{1}H NMR (400 MHz, DMSO-d_6) δ 8.93 (d, J = 8.1 Hz, 2H), 8.59 (d, J = 1.2 Hz, 2H), 8.31 (t, J = 7.6 Hz, 2H), 8.20 (d, J = 7.7 Hz, 2H), 7.98 (dd, J = 13.8, 6.1 Hz, 6H), 7.79 - 7.71 (m, 2H), 7.64 (d, J = 6.2 Hz, 2H), 7.61 - 7.50 (m, 8H), 7.06 (t, J = 7.4 Hz, 2H), 6.96 (t, J = 7.2 Hz, 2H), 6.34 (d, J = 7.5 Hz, 2H). ^{13}C NMR (126 MHz, DMSO-d_6) δ 167.33, 155.46, 150.62, 149.96, 149.14, 148.89, 143.86, 139.73, 135.52, 131.17, 130.33, 129.24, 128.79, 127.37, 125.64, 125.09, 122.26, 121.14, 116.93. HRMS (ESI, *m/z*): calcd for $C_{44}H_{32}N_4Ir$ [M-PF$_6$]$^+$ 809.2251, found 809.2255.

3, yellow solid, 171.7 mg, yield: 90%. ^{1}H NMR (400 MHz, DMSO-d_6) δ 8.88 (d, J = 8.2 Hz, 2H), 8.39 (d, J = 8.6 Hz, 2H), 8.31 (dd, J = 12.6, 6.3 Hz, 4H), 8.04 (d, J = 5.3 Hz, 2H), 8.00 (d, J = 7.7 Hz, 2H), 7.80 - 7.72 (m, 2H), 7.64 (s, 2H), 7.41 (t, J = 6.3 Hz, 6H), 7.37 (d, J = 7.8 Hz, 4H), 7.06 (t, J = 7.5 Hz, 2H), 6.95 (t, J = 7.4 Hz, 2H), 6.31 (d, J = 7.5 Hz, 2H). ^{13}C NMR (125 MHz, DMSO-d_6) δ 165.92, 155.70, 150.42, 150.04,

145.36, 143.37, 139.71, 136.88, 135.18, 134.75, 131.02, 130.50, 129.35, 128.88, 128.77, 126.28, 125.36, 122.44, 120.28. HRMS (LTQ Orbitrap XL, *m/z*): calcd for $C_{44}H_{32}N_4Ir$ $[M-PF_6]^+$ 809.2251, found 809.2251.

Preparation and Detection of TNP Samples

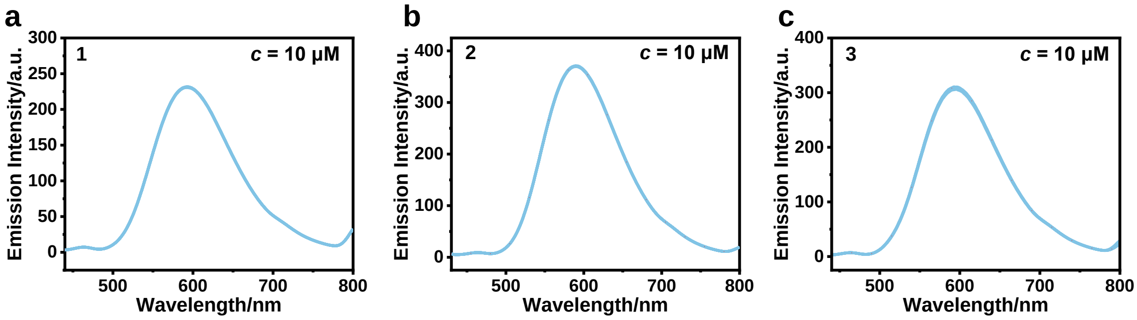

Figure S3. The emission spectra of **1**, **2**, and **3** in 11 blank samples in CH_3CN/H_2O (f_w = 60%, 10 μM).

Photophysical and AIPE properties

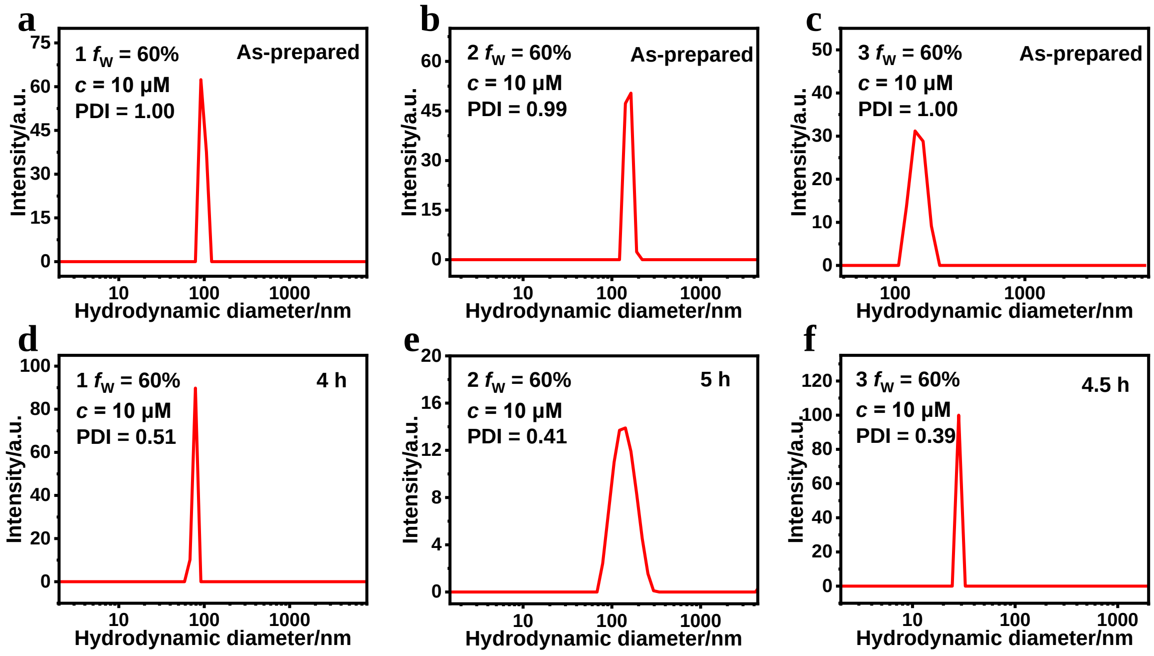

Figure S4. DLS analysis of **1**, **2**, and **3** for as-prepared and under equilibrium (10 μM, CH_3CN/H_2O).

Detection of TNP

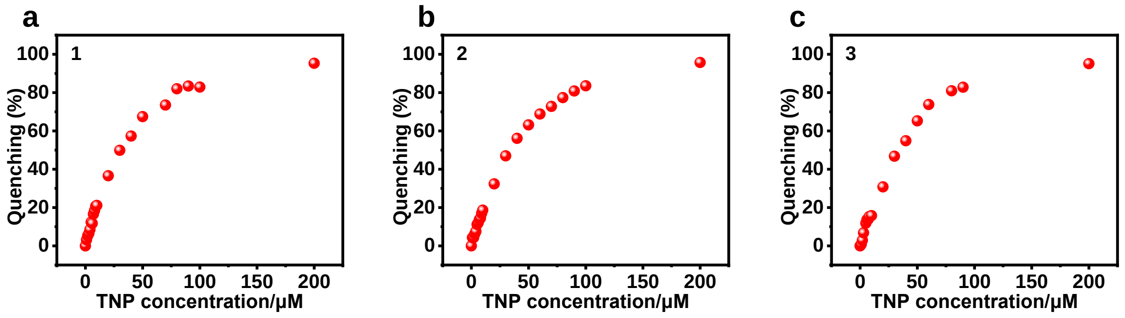

Figure S5. The quenching percentages of **1**, **2**, and **3** after addition of TNP at various concentrations.

Table S1. The emission intensity of **1**, **2**, and **3** at λ_{em} nm in 11 blank samples in CH_3CN/H_2O (f_w = 60%, 10 μM).

	λ_{em}	X_1	X_2	X_3	X_4	X_5	X_6	X_7	X_8	X_9	X_{10}	X_{11}	σ
**1**	592	231.0	230.8	230.7	231.1	231.2	231.1	231.5	231.3	231.7	231.7	231.5	0.29
**2**	591	369.3	369.6	369.9	369.8	369.9	369.7	369.6	369.8	369.9	370.2	370.9	0.41
**3**	594	309.7	310.4	309.0	310.3	309.8	309.2	308.7	308.5	308.9	309.0	309.9	0.65

Sensing Mechanism

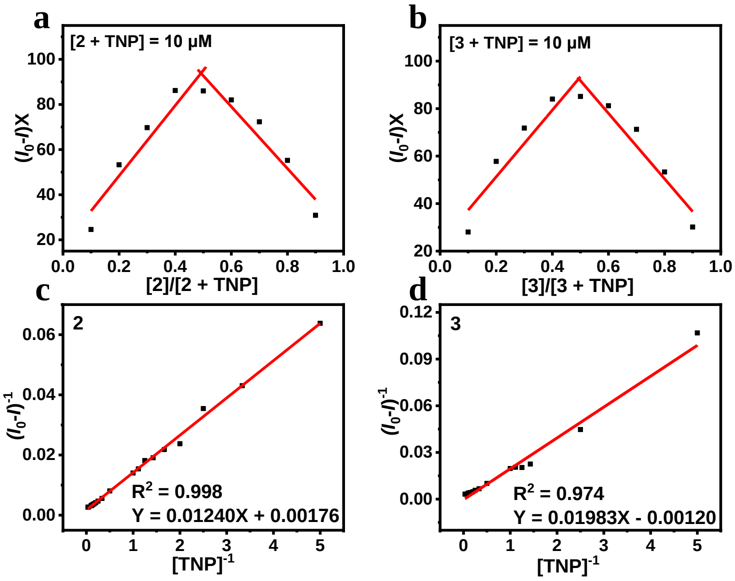

Figure S6. (a, b) The Job's plots of **2** and **3** with TNP. (c, d) Benesi-Hildebrand plots of **2** and **3** with TNP.

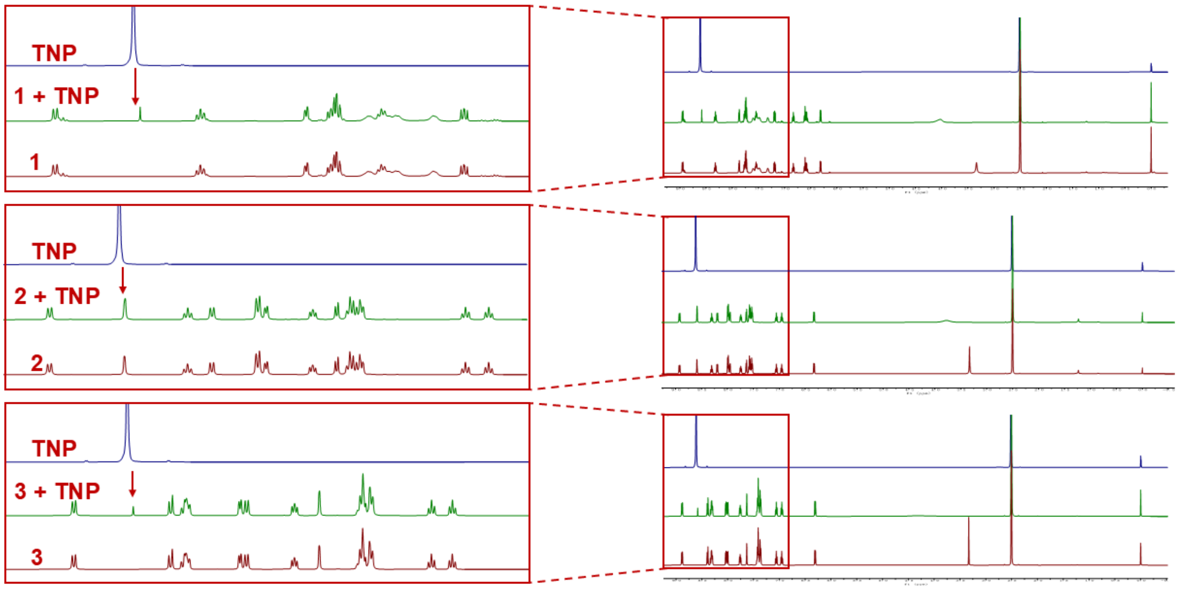

Figure S7. ^{1}H NMR spectra of **1-3**, TNP, and **1-3** + TNP.

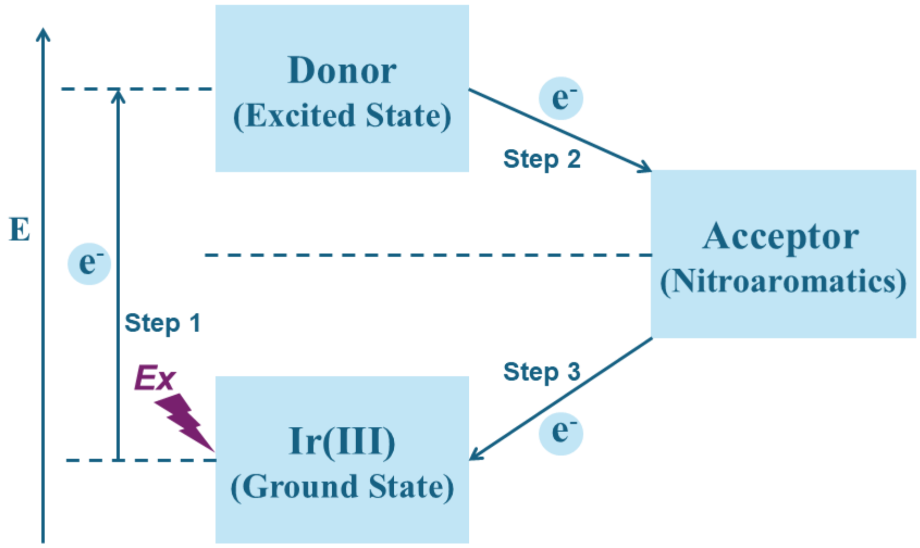

Figure S8. Diagram of the "Photo-induced Electron Transfer (PET)" process.

NMR spectra and HRMS of 1, 2, and 3

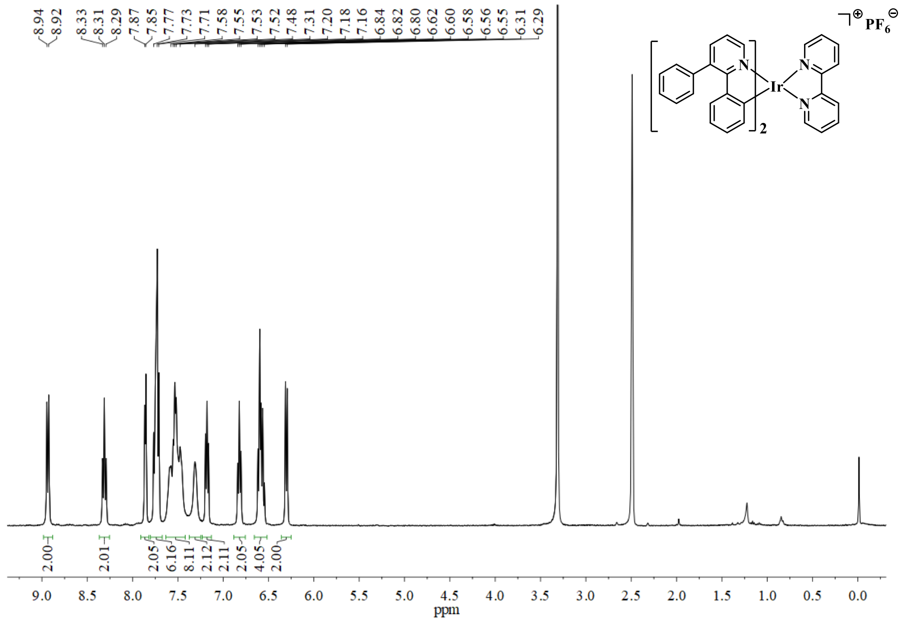

Figure S9. ^{1}H NMR spectrum of **1** in DMSO-d_6.

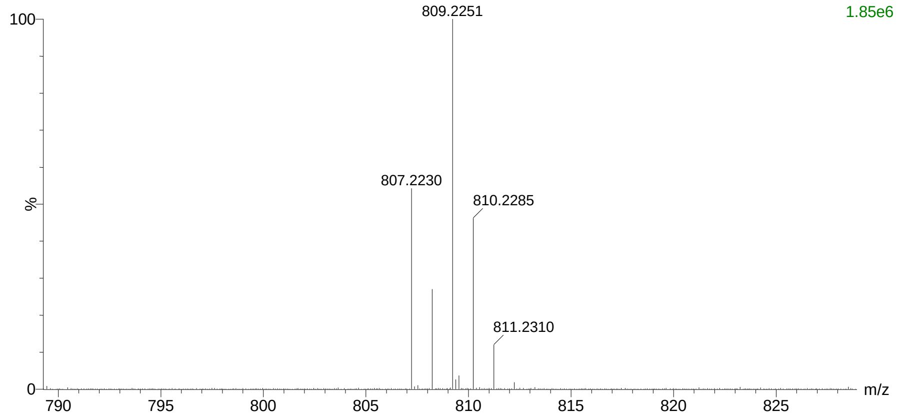

Figure S10. The HRMS of cationic portion of **1**.

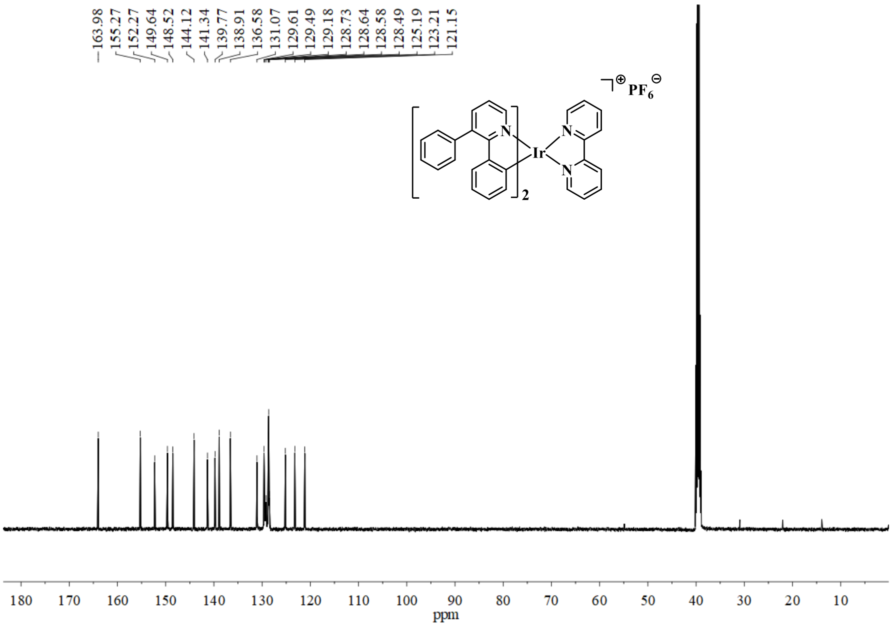

Figure S11. ^{13}C NMR spectrum of **1** in DMSO-d_6.

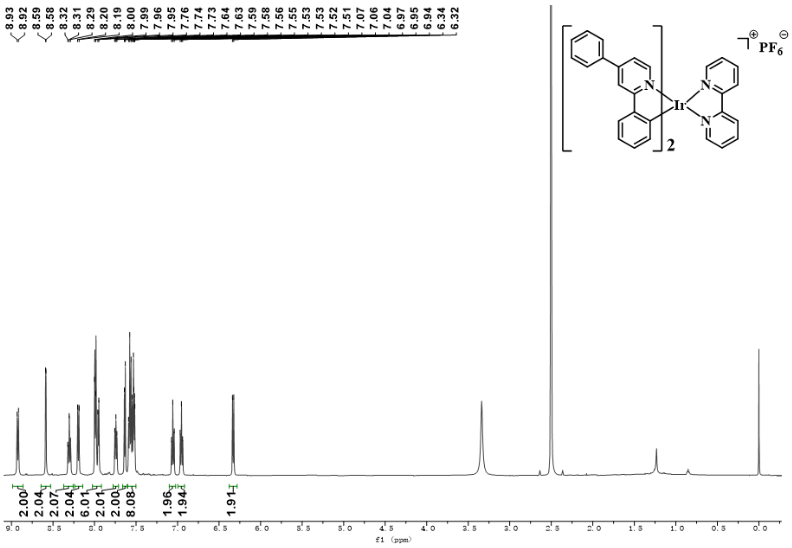

Figure S12. ^{1}H NMR spectrum of **2** in DMSO-d_6.

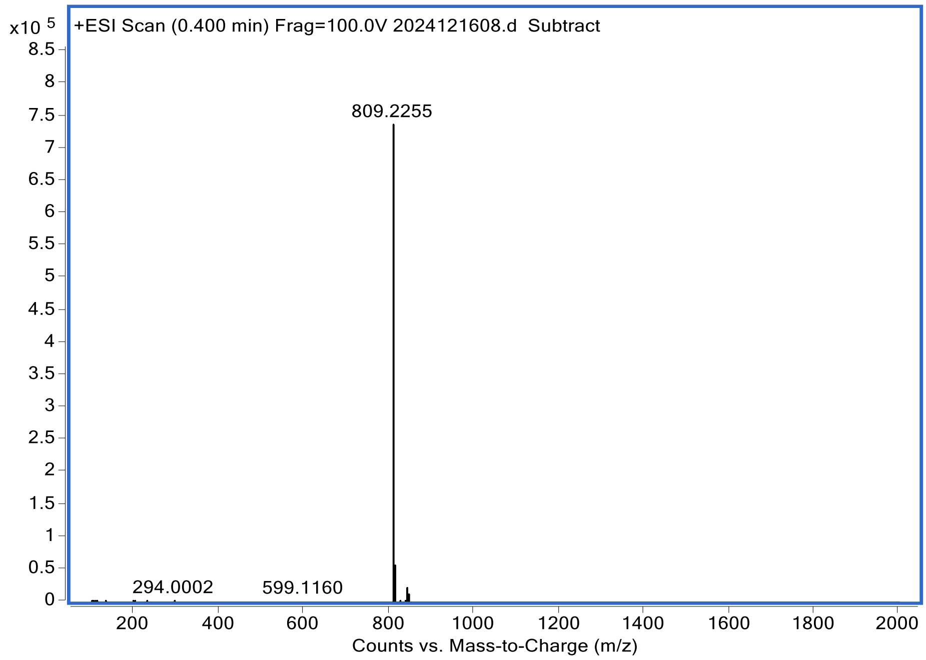

Figure S13. The HRMS of cationic portion of **2**.

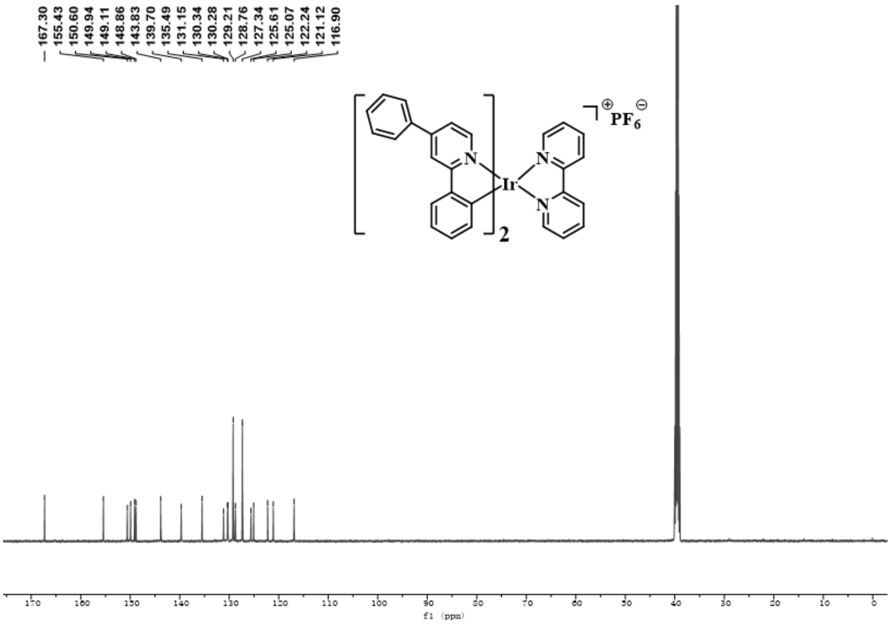

Figure S14. ^{13}C NMR spectrum of **2** in DMSO-d_6.

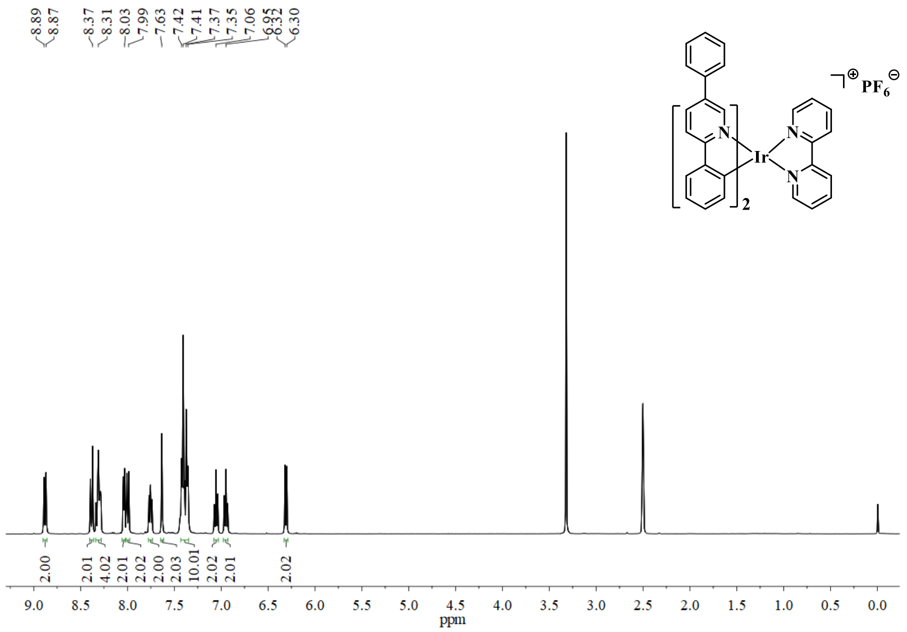

Figure S15. ^{1}H NMR spectrum of **3** in DMSO-d_6.

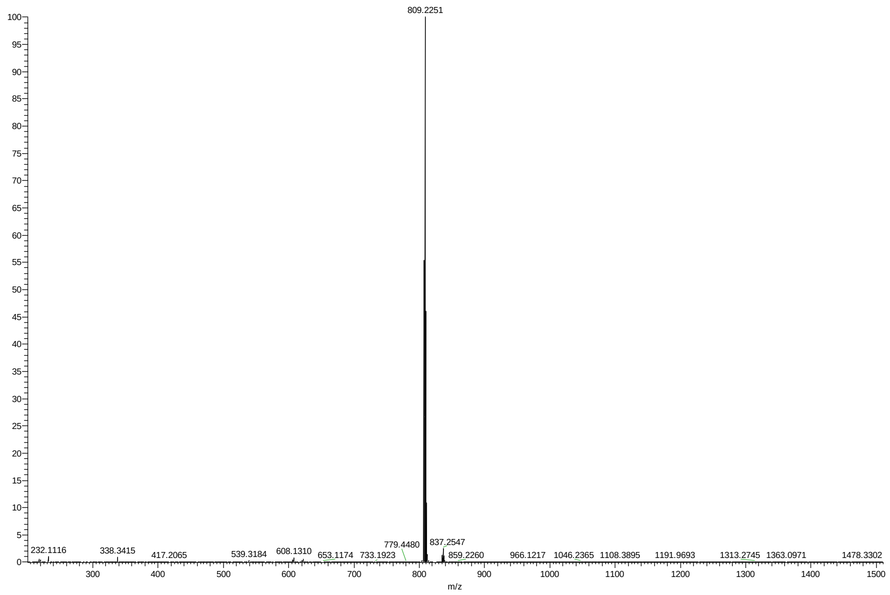

Figure S16. The HRMS of cationic portion of **3**.

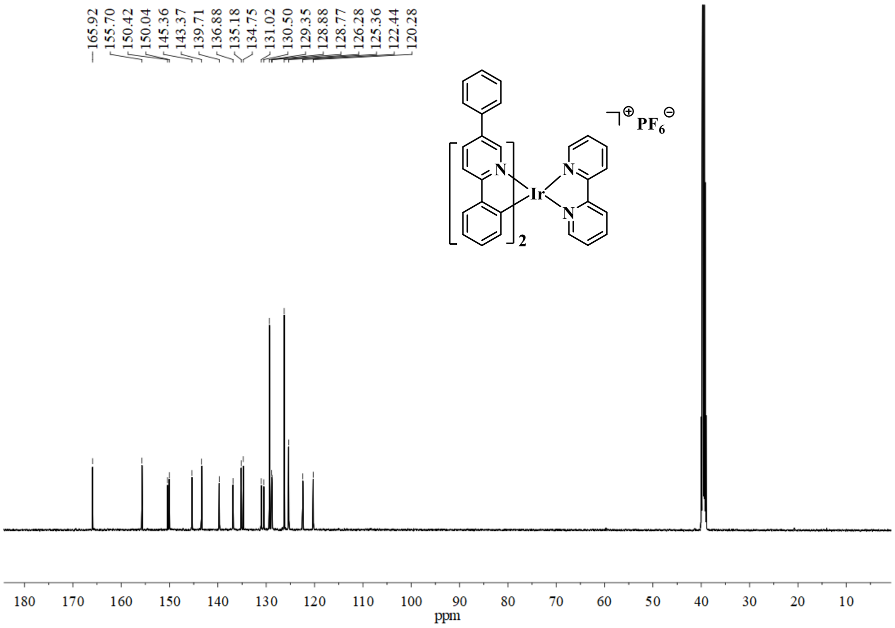

Figure S17. ^{13}C NMR spectrum of **3** in DMSO-d_6.
